# Multifunctional Periphytic Biofilms: Polyethylene Degradation and Cd^2+^ and Pb^2+^ Bioremediation under High Methane Scenario

**DOI:** 10.3390/ijms21155331

**Published:** 2020-07-27

**Authors:** Muhammad Faheem, Sadaf Shabbir, Jun Zhao, Philip G. Kerr, Shafaqat Ali, Nasrin Sultana, Zhongjun Jia

**Affiliations:** 1 State Key Laboratory of Soil and Sustainable Agriculture, Institute of Soil Science, Chinese Academy of Sciences, Nanjing 210008, China; faheem.shakoor@gmail.com (M.F.); zhaojun@issas.ac.cn (J.Z.); nasrinjc@issas.ac.cn (N.S.); 2 University of Chinese Academy of Sciences, Beijing 100049, China; 3 College of Environment, Hohai University, 1 Xikang Road, Nanjing 210008, China; sadaf.dar83@gmail.com; 4 School of biomedical Science, Charles Sturt University, Wagga Wagga, NSW 2678, Australia; philip.kerr@gmail.com; 5 Department of Environmental Sciences and Engineering, Government College University, Faisalabad 38000, Pakistan; shafaqataligill@gcuf.edu.pk; 6 Department of Biological Sciences and Technology, China Medical University, Taichung 40402, Taiwan; 7 Department of Agroforestry and Environmental Science, Sher-e-Bangla Agricultural University (SAU), Sher-e-Bangla nagar, Dhaka 1207, Bangladesh

**Keywords:** epiphyton, epixylon, methanotrophs, polyethylene, heavy metals

## Abstract

Priority pollutants such as polyethylene (PE) microplastic, lead (Pb^2+^), and cadmium (Cd^2+^) have attracted the interest of environmentalists due to their ubiquitous nature and toxicity to all forms of life. In this study, periphytic biofilms (epiphyton and epixylon) were used to bioremediate heavy metals (HMs) and to biodegrade PE under high (120,000 ppm) methane (CH_4_) doses. Both periphytic biofilms were actively involved in methane oxidation, HMs accumulation and PE degradation. Epiphyton and epixylon both completely removed Pb^2+^ and Cd^2+^ at concentrations of 2 mg L^−1^ and 50 mg L^−1^, respectively, but only partially removed these HMs at a relatively higher concentration (100 mg L^−1^). Treatment containing 12% ^13^CH_4_ proved to be most effective for biodegradation of PE. A synergistic effect of HMs and PE drastically changed microbial biota and methanotrophic communities. High-throughput 16S rRNA gene sequencing revealed that Cyanobacteria was the most abundant class, followed by Gammaproteobacteria and Alphaproteobacteria in all high-methane-dose treatments. DNA stable-isotope probing was used to label ^13^C in a methanotrophic community. A biomarker for methane-oxidizing bacteria, *pmoA* gene sequence of a ^13^C-labeled fraction, revealed that *Methylobacter* was most abundant in all high-methane-dose treatments compared to near atmospheric methane (NAM) treatment, followed by *Methylococcus*. *Methylomonas*, *Methylocystis*, *Methylosinus,* and *Methylocella* were also found to be increased by high doses of methane compared to NAM treatment. Overall, Cd^+2^ had a more determinantal effect on methanotrophic activity than Pb^2+^. Epiphyton proved to be more effective than epixylon in HMs removal and PE biodegradation. The findings proved that both epiphyton and epixylon can be used to bioremediate HMs and biodegrade PE as an efficient ecofriendly technique under high methane concentrations.

## 1. Introduction

Periphytic biofilms consist of different kinds of microorganisms including cyanobacteria, algae, diatoms, and protozoans, along with detritus, and are found either attached to submerged surfaces or floating freely in freshwater ecosystems [1,2,3]. These biofilms are classified into the following five main groups based on substrate use: epiphyton (plants), epilithon (rocks), epipelon (sediments), epixylon (wood), and epipsammon (sand). These biofilms are the constituents of phototrophic benthic microbial biofilms that play important roles as primary producers, and ultimately serve as a source of food for aquatic life [4,5,6]. They also play a crucial role in determining the degree of contamination due to their sensitivity to surrounding environmental variations and concentrations of different kinds of organic and inorganic pollutants in the aquatic ecosystem [7,8,9,10].

Generally, the microbial consortium of the periphytic biofilm has a relatively impervious nature, which tends to maintain internal processes and resist outer physicochemical variations in the surrounding environment [11,12]. Due to their robust nature, the use of periphytic biofilms as pollutant sinks does not require a lengthy acclimatization phase to adapt to local environmental conditions. Periphytic biofilms are capable of using, converting, and modifying a variety of pollutants, [13] such as microplastics [3], heavy metals [14], pharmaceutical waste [15], persistent organic pollutants, and personal care products [16].

Pollutants removal by periphytic biofilms involve the following three mechanisms: biosorption, bioaccumulation, and biodegradation [17,18,19]. Periphytic biofilms are easily acquirable, providing environmentalists with a time- and cost-efficient bioremediation system [3]. Periphytic microbes adhere to each other and to the surfaces of pollutants via extracellular polymeric substances (EPSs). These periphytic biofilms have been previously reported to be effective in the biodegradation of microplastics [20] by releasing EPSs on microplastic surfaces. In addition, they have been shown to bioremediate heavy metals (HMs) by absorbing positively charged HMs through negatively charged EPSs [21].

A report on plastic pollution speculated that, in 2050, there will be more plastic in the marine ecosystem than fish [22]. This plastic ultimately finds its way into the natural food web, accumulating in fishes and other invertebrates [23,24]. Muenmee et al. [25] found that methanotrophs are the principal plastic decomposers in the upper zone of a lysimeter, and high-density polyethylene (HDPE) plastic had the highest decay rate (k), whereas low-density polyethylene (LDPE) plastic had the lowest decay rate kinetics (k). Type I (Gammaproteobacteria) *Methylobactor* and *Methylococcus* and type II (Alphaproteobacteria) *Methylocystic* and *Methylocella* were found to be the most abundant methanotrophic species during plastic degradation [26]. Despite the huge magnitude of microplastic pollution in aquatic ecosystems, a limited amount of scientific research has focused on its eco-friendly biodegradation [3].

HMs are also persistent in the aquatic environment and can bioaccumulate in the human body, causing carcinogenic effects [27]. Similarly, Pb^2+^ and Cd^2+^ are also considered toxic and perilous to the environment [28,29,30]. Various industries such as pigments, batteries, paints, cables, steels, alloys, metal, glass, and plastic are using Pb^2+^ and Cd^2+^ extensively, thus causing aqueous pollution by dumping these metals into the aquatic ecosystem [31]. Periphytic biofilms were reported to either immobilize HMs, which means reduced bioavailability using adsorptive specific materials such as EPSs [32], or hyperaccumulate HMs from aqueous media using energy [33]. In our previous study, an epipelon biofilm was proved to be time- and cost-efficient for HMs removal under elevated methane concentrations [14]. A single culture of *Methylococcus capsulatus* (Bath) efficiently bioremediated chromium (Cr^6+^) over a wide range of concentrations (1.4−1000 mg L^−1^) [34]. However, to the best of our knowledge, few research articles are available on the bioremediation of Pb^2+^ and Cd^2+^ by periphytic methanotrophs.

Considering the capability of periphytons to remove HMs and degrade plastic from the aquatic ecosystem and the presence of a methanotrophic community in these periphytic biofilms, we aimed to evaluate the capability of periphytic biofilms to bioremediate HMs (Pb^2+^ and Cd^2+^) and biodegrade polyethylene (PE) under high methane doses. The specific aims of the current study were (1) to investigate the biodegradation of PE and bioremediation of Cd^2+^ and Pb^2+^ by two different kinds of periphytic biofilms, i.e., epiphyton (EPP) and epixylon (EPX); (2) to use the stable isotope probing (SIP) technique to study the impact of high methane dose on degradation and microbial resistance against HMs; (3) to investigate the kind of methanotrophs responsible for metal remediation and PE degradation in lake water; and (4) to assess the microbial community shift of epiphyton and epixylon biofilms as a result of their exposure to PE, Cd^2+^, and Pb^2+^.

## 2. Results and Discussion

### 2.1. SEM of Biofilms and Polyethylene Biodegradation

Epiphyton and epixylon were predominately composed of inextricably interwoven algae, bacteria, and protozoa as clearly seen in the SEM results. Prior to high methane dose treatment, fewer bacterial aggregates were visible, but after treatment with methane, bacterial aggregates were visible (Figure S1). Interwoven bundles of algae, bacteria, and phytoplankton provide space and surfaces for attachment of HMs and microplastics. The interwoven structural morphology of periphytic biofilm was found to be responsible for biodegrading microplastics and bioremediating HMs [3,14].

**Figure 1:**
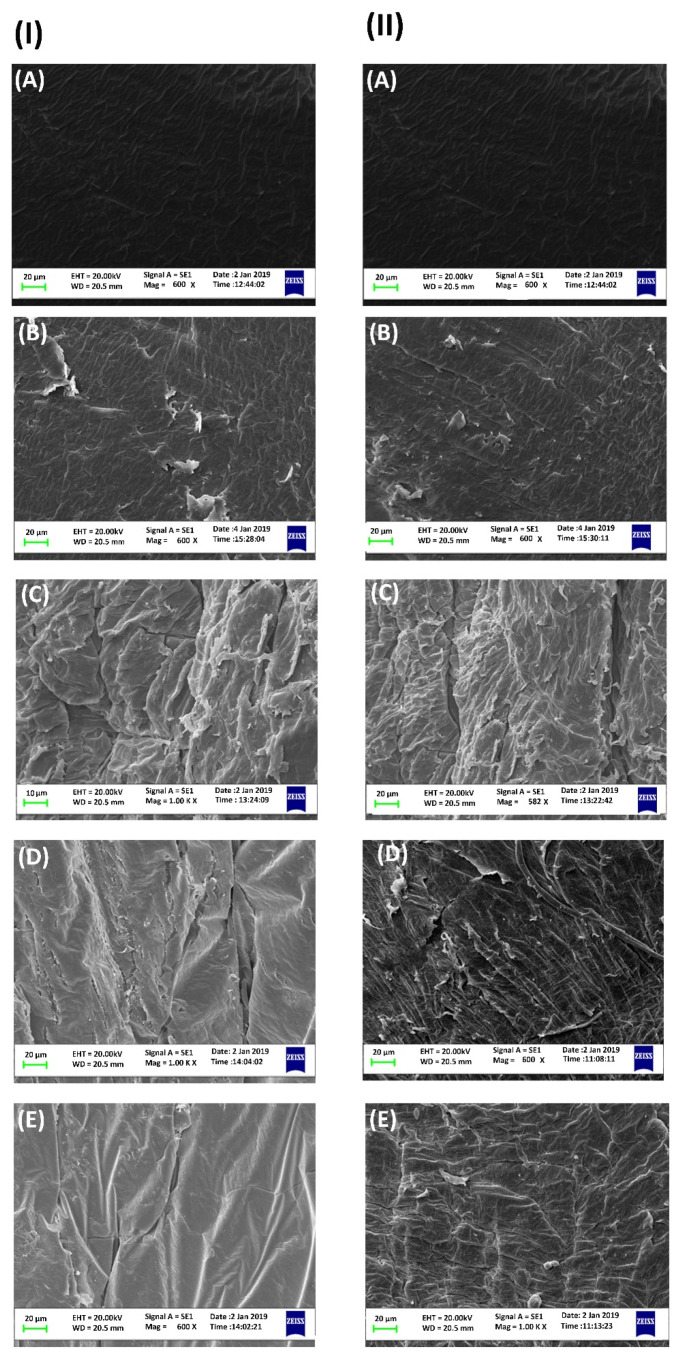
Scanning electron microscopy (SEM) micrograph at scale of 20 μm for polyethylene (PE) biodegradation (**I**) epiphyton (EPP): (**A**) control treatment,(**B**) EPP + near atmospheric methane (NAM) + PE, (**C**) EPP + ^13^C (12% methane) + PE, (**D**) EPP + ^13^C + M1C2 + PE, and (**E**) EPP + ^13^C + M2C2 + PE. (**II**) Epixylon (EPX): (**A**) control treatment, (**B**) EPX + NAM + PE, (**C**) EPX + ^13^C + PE, (**D**) EPX + ^13^C + M1C2 + PE, and (**E**) EPX + ^13^C + M2C2 + PE.

Epiphytic and epixylic biofilms were found to colonize and grow on the surface of PE pellets compared to the smooth surface of the control pellet. SEM photos of 20-day samples depicted pitted, rough, cracked, and scratchy surfaces (Figure 1). The SEM results also showed that the addition of HMs (Pb^2+^ and Cd^2+^) to the microcosm adversely affected the PE biodegradation capability of epiphyton and epixylon, but high-methane-gas amendment stimulated the biodegradation. Previously, different microbial communities were reported to grow and adhere to the surface of micro- and nano-plastic surfaces [3,35] to erode it by secreting corrosive enzymes [20]. Shabbir et al. [3] suggested that diverse microbial communities produce EPS and corrosive enzymes, which may wear away microplastic surfaces by breaking C–C structural bonds. SEM results showed a clear difference—treatments containing Pb^2+^ showed more degradation of PE compared to Cd^2+^ for both epiphyton and epixylon biofilms.

### 2.2. Microbial Activity by AWCD and Diversity Indices

The Biolog experiment showed that epiphytic and epixylic biofilms produced darker colors by feeding on different substrates revealing their higher microbial activity (Figure S1). Diversity indices of biotic integrity showed higher copiousness and abundance, efficient functional activity, richness, and evenness of microbial biota for both periphytic biofilms. For example, after seven days of incubation, the highest Shannon index was 3.69 for epiphyton and 3.50 for epixylon, which is an indicator of species evenness.

### 2.3. Biofilm Potential for Methane Oxidation under the Influence of HMs and PE

**Figure 2:**
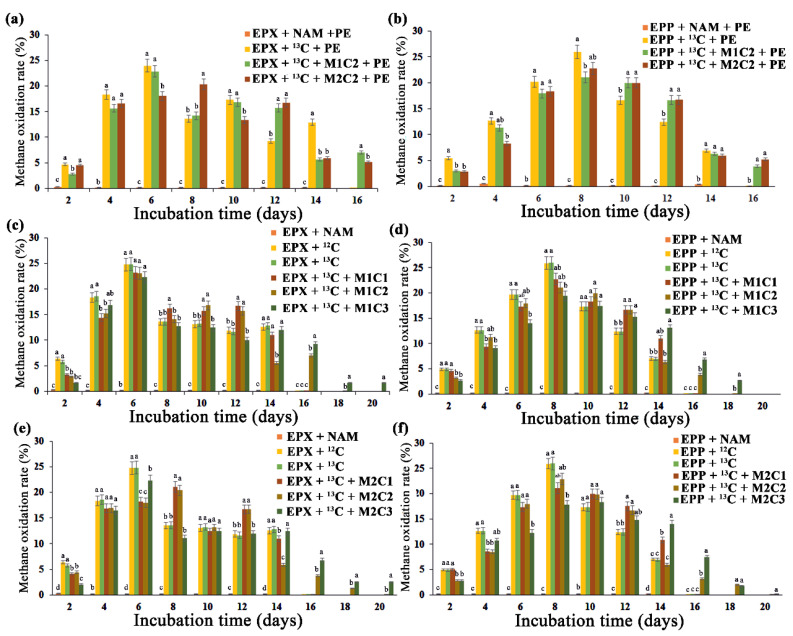
Methane oxidation rate (%) over a period of 20 days. Methane oxidation potential of (**a**) epixylon (EPX) and (**b**) epiphyton (EPP) under discrete concentrations of PE and in combination of both Pb^2+^ (M1) and Cd^2+^ (M2) at concentrations of 50 mg L^−1^ (C2) and PE. Methane oxidation potential of (**c**) epixylon and (**d**) epiphyton under different concentrations of Pb^2+^ (C1 = 2 mg L^−1^, C2 = 50 mg L^−1^, C3 = 100 mg L^−1^) along with controls. Methane oxidation potential of (**e**) epixylon and (**f**) epiphyton under different concentrations of Cd^2+^ (C1 = 2 mg L^−1^, C2 = 50 mg L^−1^, C3 = 100 mg L^−1^) along with controls. Data are expressed as mean ± standard error. The data in a column with a different letter differ significantly (*p* < 0.05) within treatments at the same day of collection based on Duncan’s test.

Epiphyton and epixylon biolfilms both showed high methane oxidation potential. The head space methane was consumed within 20 days of incubation in all the treatments. EPX + ^13^C, EPP + ^13^C and treatments with 2 mg L^−1^ of metals (Pb^2+^ and Cd^2+^) for both epiphyton and epixylon biofilms consumed headspace methane within 14 days of injection (Figure 2). Treatments with 50 mg L^−1^ of Pb^2+^ oxidized methane within 16 days of incubation, whereas the treatments with Cd^2+^ consumed methane within 18 days of incubation for both biofilms. Cd^2+^ is more lethal to periphytic biofilm compared to Pb^2+^ due to its determinantal effect on chlorophyll *a* [36]. All the treatments with 100 mg L^−1^ HMs took 20 whole days to consume headspace methane (Figure 2), which might be due to the lethal effect of high concentrations of HMs on microbial biota. The presence of a high Cd^2+^ concentration in a rice field markedly reduced periphytic biomass [37] due to its inhibitory effect on photosynthesis. In another study, marked changes in the microbial community were observed with increased Cd^2+^ and Cr^6+^ concentrations [38]. Treatments containing PE pellets, such as EPP + ^13^C + PE and EPX + ^13^C + PE treatments, oxidized headspace methane within 14 days, whereas combinations of PE with 50 mg L^−1^ of Pb^2+^ and Cd^2+^ took 16 and 18 days, respectively, to completely consume methane (Figure 2a,b). Microplastic and HMs were found to interact significantly, causing a miscellaneous change in microbial diversity, which was directly linked to methane oxidation [39]. A high dose of cadmium significantly lowered methane oxidation in soils, indicating its lethal effect on methanotrophs [40].

All high-methane-dose (12%) treatments showed high ^13^C carbon assimilation affinity derived from methane. ^13^C atom abundance was significantly enhanced from 1.08% ^13^C atoms in EPP + NAM, EPX + NAM, EPP + ^12^C and EPX + ^12^C to a maximum of 8.57% in EPP + ^13^C for epiphyton and 7.23% in EPX + ^13^C for epixylon. Treatments with HMs and PE all produced a significant increase in ^13^C atom abundance (Figure S2). Both epiphyton and epixylon biofilms showed strong methane oxidation potential by assimilating ^13^C carbon atoms, as indicated by the results.

### 2.4. Impact of Methane Oxidation and Polyethylene on Heavy Metals Bioremediation

**Figure 3:**
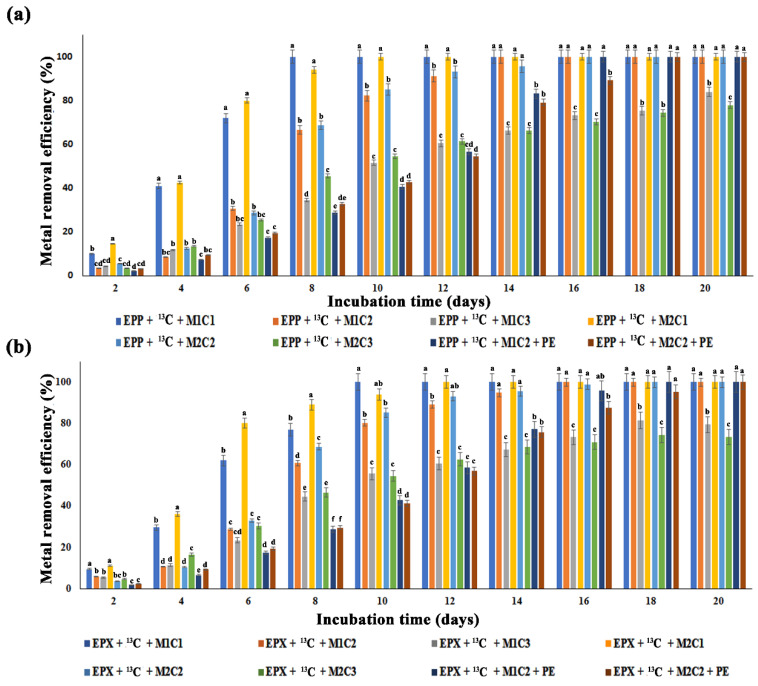
Heavy metals (HMs) removal efficiency over a period of 20 days. (**a**) Epiphyton (EPP) HMs removal efficiency under three metals concentrations (C1 = 2 mg/L, C2 = 50mg/L, C3 = 100 mg/L) of Pb^2+^ (M1) and Cd^2+^ (M2) along with a combination of PE, Pb^2+^ (M1), and Cd^2+^ (M2) at various concentrations C2 of 50 mg/L; (**b**) Epixylon (EPX) HMs removal efficiency under three metals concentrations (C1 = 2 mg/L, C2 = 50 mg/L, C3 = 100 mg/L) of Pb^2+^ (M1) and Cd^2+^ (M2) along with a combination of PE, Pb^2+^ (M1), and Cd^2+^ (M2) at concentration C2 = 50 mg/L. Data are expressed as mean ± standard error. The data in a column with a different letter differ significantly (*p* < 0.05) within treatments at the same day of collection based on Duncan’s test.

All the treatments with 2 mg L^−1^ and 50 mg L^−1^ of Pb^2+^ and Cd^2+^ completely removed HMs, but treatment with EPP + ^13^C + M1C3 only removed 84% of Pb^2+^, whereas EPP + ^13^C + M2C3 showed a 78% Cd^2+^ removal efficiency after 20 days of incubation (Figure 3a). Low efficiency was found for the treatments with 100 mg L^−1^ of HMs as due to higher metal concentration and the active sites of biofilms potentially being blocked due to fewer adsorption sites [41] and halting the continuous flow.

Similarly, EPX + ^13^C + M1C3 and EPX + ^13^C + M2C3 removed only 80.03% and 74% of Pb^2+^ and Cd^2+^, respectively (Figure 3b). Epiphyton was more efficient in removing both metals (Pb^2+^ and Cd^2+^) in terms of time duration compared to epixylon. Similarly, epiphyton took less time to completely remove Pb^2+^ compared to Cd^2+^. Treatments EPP + ^13^C + M1C1 and EPP + ^13^C + M1C2 completely removed Pb^2+^ within 8 and 14 days, respectively, whereas EPX + ^13^C + M1C1 and EPX + ^13^C + M1C2 took 10 and 16 days to remove Pb^2+^, respectively. According to Faheem et al., high methane oxidation could enhance bioremediation of Pb^2+^ and Cd^2+^ [14], and similar results were obtained in the present study—higher methane oxidation might have some positive correlation with the removal of HMs by periphytic biofilms. Treatments containing PE pellets showed two days’ delay in removing HMs, i.e., EPP + ^13^C + M1C2 + PE and EPP + ^13^C + M2C2 + PE completely remediated Pb^2+^ and Cd^2+^ in 16 and 18 days, respectively, compared to treatments without PE pellets. EPX + ^13^C + M1C2 + PE and EPX + ^13^C + M2C2 + PE adsorbed Pb^2+^ and Cd^2+^ in 18 and 20 days, respectively. Li et al. [42] found that microplastics act as a carrier and activator of HMs by increasing their bioavailability, causing a potential threat to microbiota, which is in accordance with our results.

### 2.5. GPC for Weight Loss Determination

GPC was used to evaluate the impact of metal addition and methane oxidation on the biodegradation of PE after 20 days of treatment. The results of GPC are shown in Table S1 for the samples collected on 0 days and 20 days after each treatment. We observed that the number-average molar mass (Mn) and weight-average molar masses (Mw) decreased after 20 days of treatment, signifying the biodegradation of PE by both kinds of biofilms. The most significant changes were observed in the treatments without any metal addition (~12–14%) and where 120,000 ppm methane was added. The addition of metals (Cd^2+^ and Pb^2+^) inhibited the biodegradation of PE (~3–5%), whereas Cd^2+^ addition resulted in lower biodegradation compared to Pb^2+^. Epiphyton showed better results compared to epixylon. The overall reduction in molecular weight was obvious in the values of polydispersity (Mw/Mn), which also decreased. The decrease in molecular weight is an indication of the depolymerization of long chains found in PE as well as the formation of small fragments by periphytic biofilms [43].

### 2.6. ATR-FTIR Analysis of PE Biodegradation

**Figure 4:**
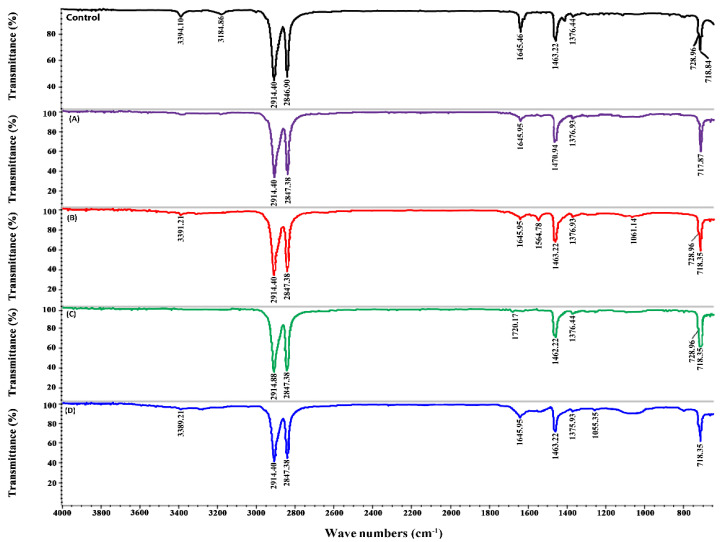
FTIR of epiphyton (EPP) showing new and vanished peaks of PE under Pb^2+^ (M1) and Cd^2+^ (M2) concentration of 50 mg/L (C2). (**A**) EPP + ^13^C + M1C2 + PE, (**B**) EPP + ^13^C + PE, (**C**) EPP + ^13^C + M2C2 + PE, and (**D**) EPP + NAM + PE.

**Figure 5:**
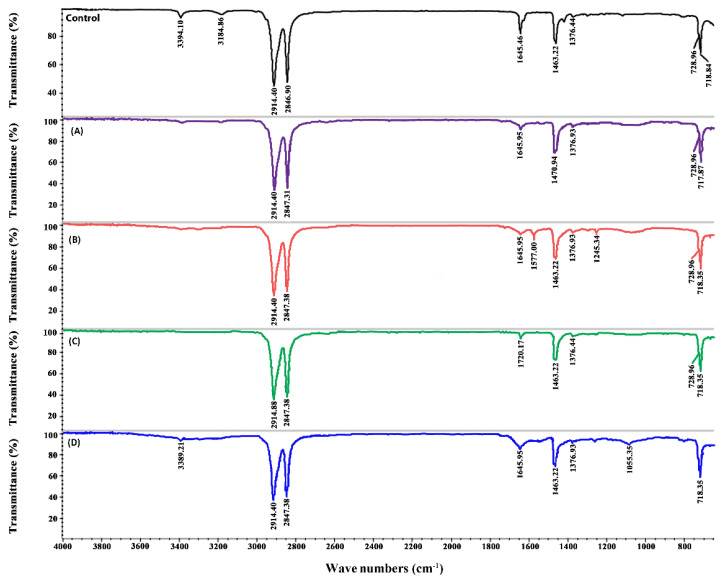
Fourier-transform infrared spectroscopy (FTIR) of epixylon (EPX) showing new and vanished peaks of PE under Pb^2+^ (M1) and Cd^2+^ (M2) concentration of 50 mg/L (C2): (**A**) EPX + ^13^C + M1C2 + PE, (**B**) EPX + ^13^C + PE, (**C**) EPX + ^13^C + M2C2 + PE, and (**D**) EPX + NAM + PE.

The spectra obtained by ATR-FTIR for different PE samples collected after 20 days showed differences in numbers and intensities of peaks (Figure 4 and Figure 5). In the control sample, the peak at 3394.14 cm^−1^ represents the O–H bond of the oxidized methylenic carbons (CH_2_). The other peaks found in the control at 2914, 2846.90, 1463, and 728 cm^−1^ represent −CH bond stretches and bends, whereas the peak at 1376 cm^−1^ represents C–H bending in the polymer structure [3]. All these peaks were also found in the treated samples; however, the intensity of the peaks was lower (Figure 4).

In the samples inoculated with epiphyton, some new peaks were seen in ATR-FTIR (Figure 4). The peaks at 3394 cm^−1^ and 3184.86 cm^−1^ disappeared in all the treatments except in EPP + NAM + PE, where 3394 cm^−1^ shifted to 3389.21 cm^−1^. In the EPP + ^13^C + PE treatment (Figure 4B), two new peaks were observed at 1577 cm^−1^ and 1245.34 cm^−1^; these peaks represent the appearance of the (C = C) and C–O stretch, respectively. Two new peaks appeared in EPX + ^13^C + PE (Figure 5B) at 1564.78 cm^−1^ and 1061.14 cm^−1^, indicating the formation of new double bonds. In the treatments where Pb^2+^ was added (Figure 4A and Figure 5A) in epixylon and epiphyton, the peak at 1463.22 cm^−1^ shifted to 1470 cm^−1^, representing the sp^2^ C‒C stretch of aromatic moieties that form during the oxidative degradation of PE. In the treatments with Cd^2+^ addition in biofilms, a new peak appeared at 1720.17 cm^−1^ (C), indicating the formation of carbonyl bond (−C = O−). The samples supplied with near atmospheric methane (D) showing the lowest weight loss had a small new peak at 1055.35 cm^−1^ representing the formation of an alkoxy group (C–O), which indicates the hydrolysis of PE [44].

Compared to epiphyton, epixylon showed a relatively lower percentage of weight loss; however, it followed the same trend of weight loss (EPX + ^13^C + PE > EPX + ^13^C + M1C2 + PE > EPX + ^13^C + M2C2 + PE > EPX + NAM + PE) (Figure 4 and Figure 5). Previously, we explained that biodegradation of PE and other microplastics by periphytic biofilms might be due to the oxidation–reduction reactions that ultimately result in the formation of a double bond structure, and carbonyl, alcoholic, and phenolic groups [3]. Similar results were obtained in different studies regarding the biodegradation of microplastics—microorganisms have the capability to degrade MPs, as observed by ATR-FTIR [45,46].

### 2.7. Methanotrophic Community Response to Heavy Metals and Polyethylene

**Figure 6:**
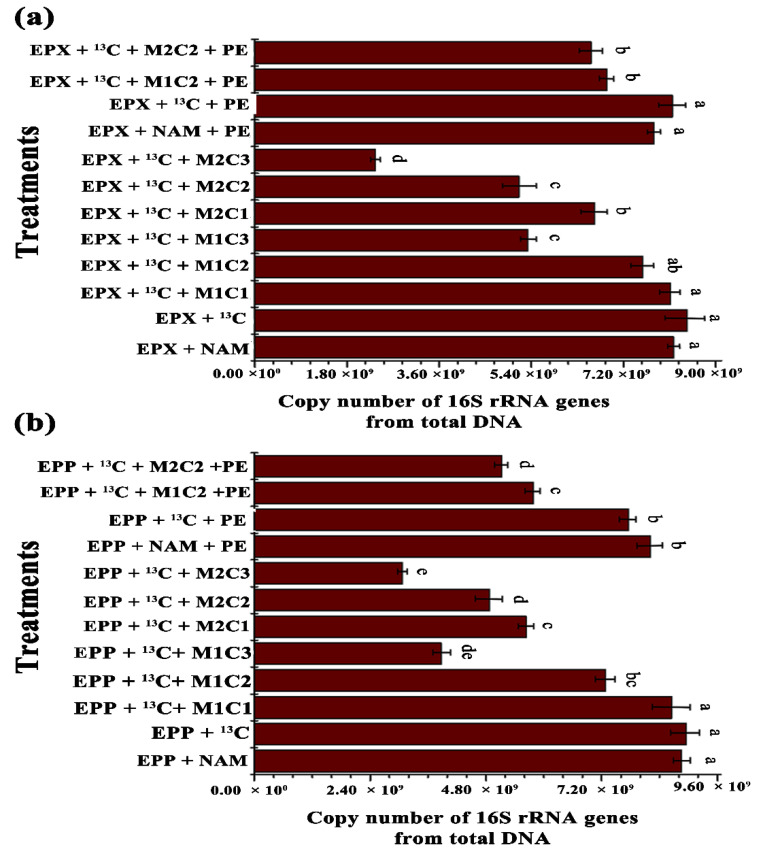
Relative abundance of 16S rRNA genes from total DNA: copy numbers of 16S rRNA genes abundance of (**a**) epixylon and (**b**) epiphyton from total DNA. The data in a column with a different letter differ significantly between treatments (*p* < 0.05) based on Duncan’s test.

Total microbiome and methanotrophic community abundance of both biofilms for all treatments were analyzed by real-time quantitative PCR of 16S rRNA and *pmoA* genes, respectively. Addition of HMs and PE alone and in combination significantly inhibited total microbiome and methanotrophic communities. EPP+ ^13^C treatment showed 1.23-, 2.30-, 1.80-, and 2.90-fold increases in 16S rRNA gene copy numbers compared to EPP + ^13^C + M1C2, EPP + ^13^C + M1C3, EPP + ^13^C + M2C2, and EPP + ^13^C + M2C3, respectively (Figure 6b). Similarly, EPX + ^13^C treatment displayed 1.11-, 1.58-, 1.63-, and 3.58-fold increases in 16S rRNA gene copy numbers compared to EPX + ^13^C + M1C2, EPX + ^13^C + M1C3, EPX + ^13^C + M2C2, and EPX + ^13^C + M2C3, respectively (Figure 6a).

EPP + ^13^C + PE produced 1.3- and 1.5-fold increases in 16S rRNA genes compared to EPP + ^13^C + M1C2 + PE and EPP + ^13^C + M2C2 + PE, respectively, whereas EPX + ^13^C + PE had 1.13- and 1.18-fold increases in 16S rRNA gene copy numbers compared to EPX + ^13^C + M1C2 + PE and EPX + ^13^C + M2C2 + PE, respectively, due to the presence of HMs and PE. The presence of PE in EPX + ^13^C + PE and EPP + ^13^C + PE reduced 16S rRNA gene copy numbers by 1.24- and 1.48-fold compared to EPP+ ^13^C and EPX+ ^13^C, respectively. Multivariate analyses clearly showed that HMs contaminants significantly reshaped the benthic microbial community composition, and decreases in 16S rRNA gene copy numbers were observed [47].

**Figure 7:**
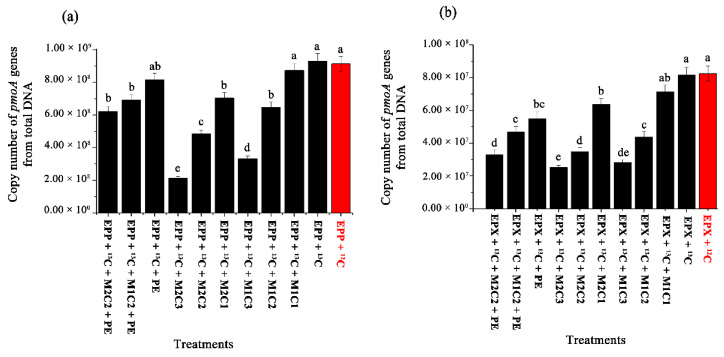
Relative abundance of *pmoA* genes from total DNA—copy numbers of *pmoA* genes abundance in (**a**) epiphyton and (**b**) epixylon from total DNA. The data in a column with a different letter differ significantly (*p* < 0.05) between treatments based on Duncan’s test.

Real-time quantitative PCR of the *pmoA* gene indicated that the presence of HMs and PE pellets had inhibitory effects on the methanotrophic community, resulting in a lower number of *pmoA* gene copy numbers. EPP+ ^13^C showed 1.44-, 2.80-, 1.92-, and 4.36-fold increases in *pmoA* gene copy number compared to EPP + ^13^C + M1C2, EPP + ^13^C + M1C3, EPP + ^13^C + M2C2, and EPP + ^13^C + M2C3, respectively (Figure 7a). The treatment involving epixylon biofilm, i.e., EPX + ^13^C, produced 1.86-, 2.87-, 2.33-, and 3.20-fold increases in *pmoA* gene copy numbers compared to EPX + ^13^C + M1C2, EPX + ^13^C + M1C3, EPX + ^13^C + M2C2, and EPX + ^13^C + M2C3, respectively (Figure 7b).

EPP + ^13^C + PE produced 1.18- and 1.31-fold increases in *pmoA* gene copy numbers compared to EPP + ^13^C + M1C2 + PE and EPP + ^13^C + M2C2 + PE, respectively, whereas EPX + ^13^C + PE showed 1.17- and 1.67-fold increases in *pmoA* gene copy numbers compared to EPX + ^13^C + M1C2 + PE and EPX + ^13^C + M2C2 + PE, respectively. Conversely, EPX + ^13^C + PE and EPP + ^13^C + PE decreased *pmoA* gene copy numbers by 1.48- and 1.14-fold compared to EPP+ ^13^C and EPX+ ^13^C, respectively. These results further verified that the presence of PE and HMs has a more adverse effect than individual augmentation, and the presence of Cd^2+^ is more harmful to bacterial communities compared to Pb^2+^. Epiphyton proved to be more resistive than epixylon in terms of 16S rRNA gene copy numbers. In our previous study, the relative abundance of *pmoA* genes in epipelon biofilm decreased in all treatment containing HMs (Cr^6+^, Pb^2+^, and Cd^2+^) compared to control [14].

### 2.8. Identification of Methanotrophic Activity by SIP

**Figure 8:**
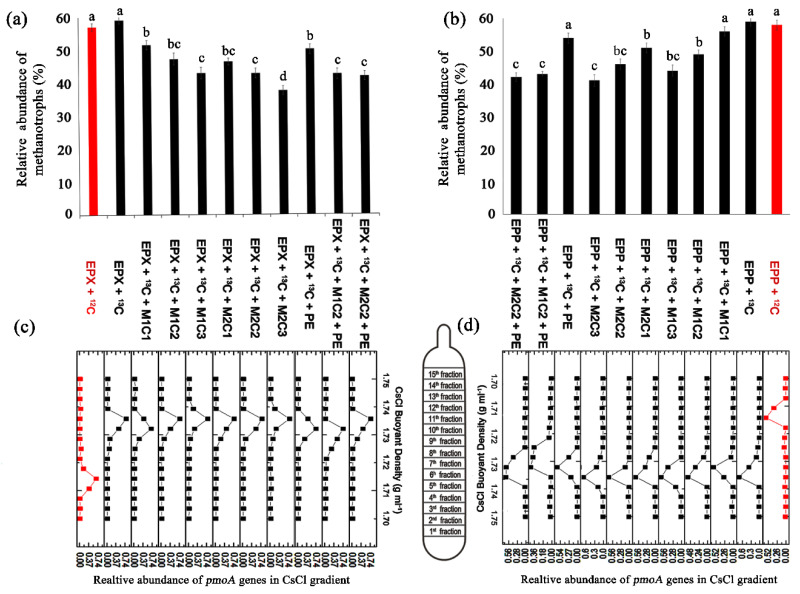
Relative abundances of *pmoA* genes of the methane-derived heavy fraction, calculated from each fraction to the total abundance throughout the treatment. Red columns represent the light fraction and black columns represent heavy fractions: ^13^C-methane-derived *pmoA* genes abundance from heavy fraction of (**a**) epixylon and (**b**) epiphyton. Gradient distribution of heavy (buoyant density around 1.735) and light (buoyant density around 1.715) fractions in (**c**) epixylon and (**d**) epiphyton. The data in a column with a different letter differ significantly (*p* < 0.05) between treatments based on Duncan’s test.

The heavy fraction (containing ^13^C) obtained from isopycnic centrifugation of SIP DNA was subjected to QPCR and clearly indicated active methanotrophic cell propagation in all high methane dose treatments, except for NAM treatments, which showed no significant active propagation. A peak shift of relative *pmoA* abundance of the heavy fraction was clearly visible around a buoyant density of 1.735 g mL^−1^ in all high methane dose treatments for both epiphyton (Figure 8c) and epixylon (Figure 8d) biofilms. The light fraction ^12^C *pmoA* gene abundances peaked around buoyant density of 1.715 g mL^−1^ and were only detectable in EPP + ^12^C and EPX + ^12^C treatments.

EPP + ^13^C + M1C1, EPP + ^13^C + M1C2, EPP + ^13^C + M1C3, EPP + ^13^C + M2C1, and EPP + ^13^C + M2C2, and EPP + ^13^C + M2C3 demonstrated 3%, 10%, 15%, 5%, 13%, and 18% less propagation in the presence of HMs at low to high concentration, respectively, compared to EPP + ^13^C (Figure 8b).

Epixylon treatments EPX + ^13^C + M1C1, EPX + ^13^C + M1C2, EPX + ^13^C + M1C3, EPX + ^13^C + M2C1, EPX + ^13^C + M2C2, and EPX + ^13^C + M2C3 also showed 7.53%, 11.79%, 16.11%, 12.71%, 16.29%, and 21.47% fewer copy numbers of the *pmoA* gene than EPX + ^13^C, respectively (Figure 8a). Similarly, the presence of PE alone and in combination with HMs in both epiphyton and epixylon biofilms reduced the methanotrophic activity. EPP + ^13^C + PE, EPP + ^13^C + M1C2 + PE, and EPP + ^13^C + M2C2 + PE had 5%, 17%, and 18% fewer copy numbers of *pmoA* genes compared to control treatment (EPP + ^13^C), respectively. Within epixylon treatments, *pmoA* gene copy number was also reduced to 9%, 16.50%, and 17.19% in EPX + ^13^C + PE, EPX + ^13^C + M1C2 + PE, and EPX + ^13^C + M2C2 + PE, respectively, compared to the epixylon control (EPX + ^13^C). From the results of QPCR of *pmoA*, we further confirmed that copy numbers of 16S rRNA gene were also reduced due to the determinantal effect of HMs and PE. Brennecke et al. [39] found that HMs in the presence of microplastics pose serious threats to marine microbial life. Cd^2+^ proved to be more toxic to methanotrophic communities than Pb^2+^. Methane oxidizers presented relatively discrete metal resistance patterns by showing resistance to different metals but proved to be sensitive to cadmium [48]. Methanotrophic communities were reported to be reduced in paddy soils, which was also due to cadmium contamination [49].

### 2.9. Epiphyton Microbial Community Response to Methane, Heavy Metals, and Polyethylene

**Figure 9:**
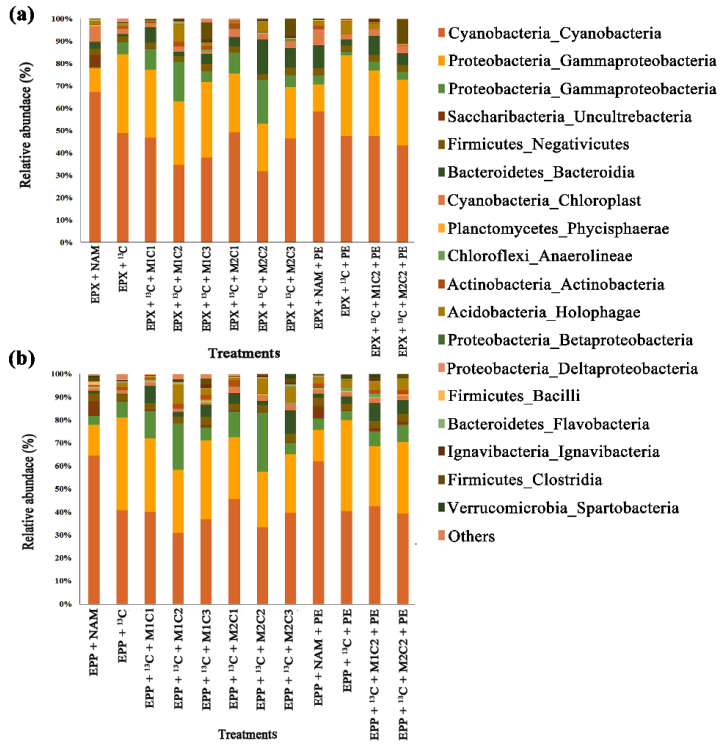
High-throughput 16S rRNA gene sequencing from total DNA showing the relative abundance of different phylum and classes in (**a**) epixylon and (**b**) epiphyton of all low and high methane treatments derived from ^13^C fraction of DNA obtained by gradient centrifugation of treatments with Pb^2+^ (M1) and Cd^2+^ (M2) at concentration of 50 mg L^−1^ (C2) and in combination with PE.

High-throughput 16S rRNA gene sequencing of epiphytic biofilms showed that all the epiphytic treatments with high methane doses severely changed the microbiome under the effect of HMs and PE pellets. Cyanobacteria was the most abundant class followed by Gammaproteobacteria and Alphaproteobacteria among all treatments. Treatment EPP + ^13^C had 1.13%, 8.65%, 13.48%, 2.14%, 7.17%, and 9.52% more OTUs of Cyanobacteria than EPP + ^13^C + M1C1, EPP + ^13^C + M1C2, EPP + ^13^C + M1C3, EPP + ^13^C + M2C1, EPP + ^13^C + M2C2, and EPP + ^13^C + M2C3, respectively (Figure 9b). Epiphyton treatment with PE, EPP + ^13^C + PE had 4.24% and 6.81% more OTUs compared to EPP + ^13^C + M1C2 + PE and EPP + ^13^C + M2C2 + PE, respectively.

Increases in proteobacterial abundance represent their importance in bioremediation of HMs and in microplastics biodegradation. Proteobacteria was the most abundant class for the bioremediation of HMs [50]. Microplastics act as sources of HMs and plastic additives, which together, enhance the determinantal effect [51].

Gammaproteobacterial OTUs were reduced by 9.36%, 12.32%, 15.27%, 16.62%, 17.50%, and 22.10% in EPP + ^13^C + M1C1, EPP + ^13^C + M1C2, EPP + ^13^C + M1C3, EPP + ^13^C + M2C1, EPP + ^13^C + M2C2, and EPP + ^13^C + M2C3 treatments, respectively, compared to EPP + ^13^C due to the presence of HMs. EPP + ^13^C + PE had 13.72% and 13.35% more OTUs than EPP + ^13^C + M1C2 + PE and EPP + ^13^C + M2C2 + PE, respectively. A number of studies proved the role of Gammaproteobacteria in bioremediation of HMs and biodegradation of plastics [52,53].

Unlike Gammaproteobacteria, Alphaproteobacteria was favored by the presence of HMs, with treatments EPP + ^13^C + M1C1, EPP + ^13^C + M1C2, EPP + ^13^C + M2C1 and EPP + ^13^C + M2C2 having 5.10%, 15.51%, 3.82% and 20.03% more OTUs compared to EPP + ^13^C, respectively. However, the high dose of HMs (100 mg L^−1^) adversely effected Alphaproteobacteria in EPP + ^13^C + M1C3 and EPP + ^13^C + M2C3 treatments by reducing its OTUs to 2.85% and 3.19%, respectively, compared to EPP + ^13^C. Alphaproteobacteria and Gammaproteobacteria were found to rapidly colonize the surface of marine plastics [54]. Pyrosequencing of sites with HMs contamination revealed that Alphaproteobacteria were more resistant to polyaromatic hydrocarbons (PAHs) and HMs [55].

### 2.10. Epixylon Microbial Community Response to Methane, Heavy Metals, and Polyethylene

Based on 16S rRNA gene sequence results, epixylon followed the same pattern of microbial community response as epiphyton. Epixylic treatments with a high methane dose, HMs, and PE pellets also severely affected the microbial diversity of epixylon biofilms, which was dominated by phylum and/or class Cyanobacteria followed by class Gammaproteobacteria, Alphaproteobacteria, Saccharibacteria, etc. Treatment EPX + ^13^C had 3.13%, 18.10%, 23.47%, 5.27%, 17.31%, and 13.65% more OTUs of Cyanobacteria than EPX + ^13^C + M1C1, EPX + ^13^C + M1C2, EPX + ^13^C + M1C3, EPX + ^13^C + M2C1, EPX + ^13^C + M2C2, and EPX + ^13^C + M2C3, respectively (Figure 9a). Treatment with PE, EPX + ^13^C + PE had 9.23% and 16.83% more cyanobacterial OTUs compared to EPX + ^13^C + M1C2 + PE and EPX + ^13^C + M2C2 + PE, respectively. Epixylon was less resistant to HMs than epiphyton. Among the treatments containing PE, EPX + ^13^C + PE had 9.23% and 13.68% more OTUs than EPX + ^13^C + M1C2 + PE and EPX + ^13^C + M2C2 + PE, respectively, indicating HMs along with PE produce more harmful effects on epixylon biofilms compared to epiphyton biofilms.

Similar to epiphyton, epixylon also showed a lower number of OTUs for Gammaproteobacteria, i.e., 6.36%, 9.32%, 10.95%, 13.62%, 14.64%, and 20.09% reductions in EPX + ^13^C + M1C1, EPX + ^13^C + M1C2, EPX + ^13^C + M1C3, EPX + ^13^C + M2C1, EPX + ^13^C + M2C2 and EPX + ^13^C + M2C3 treatments, respectively, compared to EPX + ^13^C. Under the effect of PE and HMs EPX + ^13^C + M1C2 + PE and EPX + ^13^C + M2C2 + PE had 20.74% and 14.34% lower numbers of OTUs compared to EPX + ^13^C + PE, respectively.

Epixylic Alphaproteobacteria results further confirmed the detrimental effect of HMs, as was observed in epiphytic Alphaproteobacteria as well. EPX + ^13^C + M1C1, EPX + ^13^C + M1C2, EPX + ^13^C + M2C1, and EPX + ^13^C + M2C2 treatments propagated well and had 5.09%, 15.41%, 5.87%, and 15.20% more OTUs compared to EPX + ^13^C, respectively. Treatments EPX + ^13^C + M1C3 and EPX + ^13^C + M2C3 had 1.95% and 1.33% lower numbers of OTUs compared to EPX + ^13^C, respectively.

### 2.11. Methanotrophic Community Response to Methane, Heavy Metals, and Polyethylene

Based on complete removal of HMs and PE biodegradation under high methane doses, heavy fractions of control treatments of both biofilms (containing only 12% of methane), treatments containing 50 mg L^−1^ of both HMs alone and in combination with PE along with control of PE (containing 12% methane and PE) were subjected to *pmoA* gene sequencing for the identification of active methanotrophs responsible for Pb^2+^, Cd^2+^ removal, and PE biodegradation.

**Figure 10:**
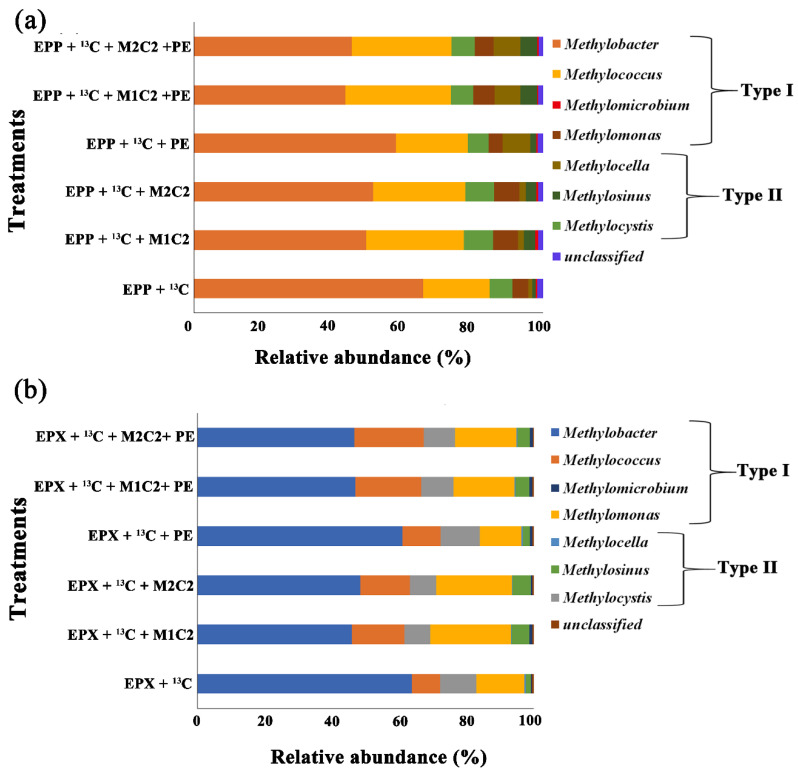
High-throughput *pmoA* gene sequencing showing relative abundance of pmoA in (**a**) epiphyton and (**b**) epixylon derived from ^13^C fraction of DNA obtained by gradient centrifugation of treatments with Pb^2+^ (M1) and Cd^2+^ (M2) at a concentration of 50 mg L^−1^ (C2) and in combination with PE.

Sequence results of ^13^C fraction *pmoA* genes indicated that Type I methanotrophs outnumbered Type II in all high methane dose treatments. Type I *Methylobacter* sp. was the most abundant species in all treatments, but in the presence of HMs, methanotrophic abundance reduced by 16.30%, 14.28%, 7.82%, 22.27%, and 22.18% in EPP + ^13^C + M1C2, EPP + ^13^C + M2C2, EPP + ^13^C + PE, EPP + ^13^C + M1C2 + PE, and EPP + ^13^C + M2C2 + PE, respectively, compared to EPP + ^13^C (Figure 10a). *Methylobacter* Type I was the most dominant in all applied treatments for HMs bioremediation [14]. Unlike *Methylobacter* Type I, the relative abundance of *Methylcoccus* was higher in EPP + ^13^C + M1C2, EPP + ^13^C + M2C2, EPP + ^13^C + PE, EPP + ^13^C + M1C2 + PE, and EPP + ^13^C + M2C2 + PE by 8.99%, 7.33%, 1.63%, 11.23%, and 12.37, respectively. *Methylococcus* have been widely used as single and/or mix cultures for bioremediation of HMs such as lead and chromium [14,34]. The presence of HMs and PE stimulated *Methylococcus*, *Methylocystis*, and *Methylosinus*. *Methylococcus capsulatus* was found at a depth of 15 cm in soil and was efficient in the biodegradation of plastics [25]. *Methylocystis*, *Methylocella*, and *Methylosinus* were already reported to bioremediate a HM-contaminated site and to biodegrade microplastics [26].

Taxonomic analysis of epixylon ^13^C-labelled methanotrophic gene revealed a distinct type of methanotrophic species. *Methylobacter* Type I was the most abundant species in all high methane dose treatments, similar to the epiphytic biofilm. *Methylobacter* relative abundance was lowered by the presence of HMs and PE by 17.95%, 15.41%, 2.87%, 16.86%, and 17.14% in EPX + ^13^C + M1C2, EPX + ^13^C + M2C2, EPX + ^13^C + PE, EPX + ^13^C + M1C2 + PE, and EPX + ^13^C + M2C2 + PE, respectively, compared to EPX + ^13^C (Figure 10b). *Methylobacter* were found to be responsible for the biodegradation of plastic in a semi-aerobic landfill [26]. *Methylococcus*, *Methylomonas,* and *Methylosinus* gene abundance was higher in the presence of HMs and PE in all treatments except for EPX + ^13^C, but abundance of *Methylocystis* was lower compared to EPX + ^13^C. *Methylomonas* sp. DH-1 has been widely used in the production of acetone [56], which is a well-known solubilizing agent for plastics.

## 3. Materials and Methods

### 3.1. Microplastic and Heavy Metals

The PE used in this study was 1–4 mm diameter spheroids in the form of pellets, which were mechanically resized to obtain 1000 µm pellets. The structure and properties of PE are described in Table S2. HM salts PbCl_2_ and CdCl_2_ were used for Pb^2+^ and Cd^2+^, respectively. The stock solutions (each 500 mg L^−1^) of metal salts were prepared using deionized water and diluted accordingly. All the chemicals and PE were purchased from Sigma-Aldrich (Saint Louis, MO, USA) and were of analytical grade.

### 3.2. Isolation and Growth Conditions of Epiphytic and Epixylic Biofilms

Epiphyton (from the base of water lily flower plant) and epixylon (from the base of dock wooden poles) were isolated from Xuan Wu Lake, Nanjing, China, using standard isolation methods. The periphytic biofilms were transported to the laboratory under low temperature (4 °C) and were grown separately using modified woods hole culture (WC) media, as described previously [14]. Briefly, both periphytic biofilms were individually grown at 28 °C using a 16 h light/8 h dark period in 5 L glass beakers, and the pH was adjusted to 6.8. After 70 days in the growth chamber, 5 mL of each suspended biofilm along with 15 mL of modified WC (without carbon source) were incorporated to each microcosm (120-mL serum bottles) separately according to the experimental design. All serum bottles were kept in the growth chamber for 20 more days to stabilize the growth of periphytic biofilms. Subsequently, methane gas was injected into microcosms along with HMs and PE pellets for further study.

### 3.3. Sampling Sites’ Physicochemical Properties

The sampling sites for both epiphyton and epixylon biofilms were tested for their physicochemical properties, i.e., epiphyton: nitrate 0.69 mg L^−1^, ammonia 0.59 mg L^−1^, total nitrogen (TN) 2.10 mg L^−1^, total phosphorus (TP) 0.21 mg L^−1^, and pH 7.74; epixylon: nitrate 0.74 mg L^−1^, ammonia 0.63 mg L^−1^, TN 2.14 mg L^−1^, TP 0.25 mg L^−1^, and pH 7.79.

### 3.4. Morphology and Activities of Periphytic Biofilm and Polyethylene

The morphological changes in epiphyton and epixylon biofilms were determined before and after treatment with a high methane dose using scanning electron microscopy (SEM; EVO 18, Zeiss, Oberkochen, Germany). Similarly, morphological changes in PE were also observed under SEM before and after treatment with a high methane dose and HMs. The Biolog™ plate technique (Agilent, Santa Clara, CA, USA) with different substrates was employed to assess the microbial activities of biofilms, as previously described [14]. Briefly, 2 g of biofilm suspension was centrifuged at 12,000 rpm and 25 °C. Pellets obtained after centrifugation were resuspended in plate wells and the plate was incubated and observed at 25 °C for the average well color development (AWCD) through a Biolog™ Microplate Reader ELx808 at 590 nm (Agilent, Santa Clara, CA, USA) for seven consecutive days. Diversity indices of biotic integrity were calculated.

### 3.5. Experimental Setup for DNA-Stable Isotopes Probing

We studied metals bioremediation and PE biodegradation in microcosms (sterile 120 mL serum bottles) containing 20 mL of suspended material (5 mL of suspended biofilms and 15 mL of modified WC medium), as previously described [14]. A series of different concentrations (2, 50, and 100 mg L^−1^) of HMs (Pb^2+^, Cd^2+^) was employed in quintuplicate to these microcosms to assess the bioremediation potential of epiphyton and epixylon biofilms.

To evaluate the capability of biofilms to biodegrade microplastic, five replicates of each treatment were studied using a microcosm containing 5 mL of each biofilm and 15 mL of modified WC along with a pellet of PE. To determine the effect of HMs on the biodegradation potential and microbial diversity under high methane doses, four separate treatments containing HMs including (50 mg L^−1^ each of Pb^2+^ and Cd^2+^), biofilms, and PE pellets were prepared. After the preparation of biofilm-treatment mixtures, microcosms were sealed with butyl stoppers and headspace ^13^CH_4_ and ^12^CH_4_ gases were employed to each microcosm. NAM concentration for low methane dose treatments and 12% methane for high methane dose treatments were used for all prepared treatments. The detailed experimental design is described in Tables S3 and S4. The remaining volume of microcosm was filled with N_2_ and O_2_ according to atmospheric volumetric ratios.

All microcosms were placed in a shaker incubator at 150 rpm with 16 h of light and 8 h of dark at 28 °C and pH was adjusted to 6.8, which is optimum for biofilm growth. The ^13^CH_4_ (>99% ^13^C-atom pure) was obtained from Cambridge Isotope Laboratories (Tewksbury, MA, USA). Headspace CH_4_ concentration was measured using gas chromatography pro-2010 (Shimadzu GC12-A, Nagara, Japan) every 48 h. The incubation was stopped when the change in methane concentration was undetectable. PE samples were collected after changes in methane concentration were no longer detectable. Biofilms were collected from each treatment and stored at −20 °C for further investigation. For ^13^C-atom relative abundance, 1 g of vacuum-freeze-dried sample of each treatment was subjected to a Flash 2000 elemental analyzer (Thermo Scientific, Waltham, MA, USA) attached to a Delta V™ IRMS Advantage isotope mass ratio spectrometer (Thermo Scientific, USA). Inductively coupled plasma mass spectrometry (ICP-MS) was used to measure the concentrations of metals (Pb^2+^ and Cd^2+^). To avoid HMs precipitation, a few drops of concentrated HNO_3_ were added to avoid precipitation before metals detection.

### 3.6. Determination of Weight Loss of PE

PE pellets were removed from the microcosms after 20 days of incubation with methane level ≤100 ppm and biodegradation by epiphyton and epixylon. Biofilm constituents were removed from pellets by deionized water and washed with ethanol. The pre-weighed PE pellets were weighed using Mettler Toledo AL54 four-decimal-place analytical lab balance (±0.00005 g; Shanghai, China). The percentage weight loss (%) for all treatments was calculated by the following equation (Equation (1)), as described earlier:

(1) $$Percent\;weight\;loss=\left(\frac{W˳-W_{t}}{W˳}\right)\times 100,$$

where *W˳* is the initial weight of the PE pellet and *W_t_* is the weight of PE after biodegradation.

### 3.7. Structural Analysis by FTIR and Gel Permeation Chromatography (GPC)

PE polymeric structural change after biodegradation under a high methane dose was recorded by Fourier-transform infrared spectroscopy coupled with attenuated total reflectance (FTIR–ATR; Nicolet 8700, Thermo Electron Co., Waltham, MA, USA) with a scanning power with a resolution of 4 cm^−1^ and a 500–4000 cm^−1^ range. PE samples from all treatments including the control (non-inoculated sample) were subjected to FTIR analysis.

PE biodegradation was further confirmed through gel permeation chromatography (GPC) by evaluating the molecular weight changes. A PL-GPC PL50 (Agilent, Santa Clara, CA, USA) system coupled with the triple mixed-b columns and an RI detector was used for this purpose.

PE was solubilized in 1,2,4-tricholobenzene (TCB) and 0.15 butylated hydroxytoluene (BHT) at 160 °C and the same chemicals were used as eluents in GPC. The eluent flow rate was maintained at 1 mL min^−1^ with a column temperature of 160 °C. Agilent chemstation software A.10.02 (Agilent, Santa Clara, CA, USA) was used for data analysis and Agilent (Agilent, USA) polystyrene standards were used for calibration curves.

### 3.8. Isopycnic Fractionation and Quantification of pmoA Gene

The fast DNA spin kit for soil (MP Biomedicals, Irvine, CA, USA) was used to extract whole DNA from 500 mg biofilm samples from each treatment according to the manufacturer’s instructions. The quantity of extracted DNA was assessed by a NanoDrop ND-1000 UV-visible light spectrophotometer (NanoDrop Technologies, Wilmington, DE, USA). Density gradient isopycnic centrifugation of total DNA was employed to isolate ^12^CH_4_ from ^13^CH_4_ fractions of all treatments, as described earlier [57]. Polyethylene glycol 6000 (PEG 6000) was used to precipitate heavy and light fractions of DNA from the CsCl medium and diluted to 25 µL using an elution buffer for downstream analysis.

Methanotrophic biomarker *pmoA* genes from total DNA and fractionated DNA (1–15) were quantified using a PCR CFX96 Optical Real-Time Detection System (Bio-Rad, Hercules, CA, USA) to ascertain the abundances of methane oxidizing bacteria (MOB) and incorporation of ^13^C into methanotrophic communities of biofilms. Thermal cycling conditions and the primer pair used are detailed in Table S5. PCR reaction mixtures obtained from Sigma-Aldrich (USA) were used to prepare standards as reported previously [58]. The achieved amplification efficiency was 94% to 99%, with *R*^2^ values of 0.996 to 0.999.

### 3.9. MiSeq Sequencing of Total DNA and Heavy Fractions

16S rRNA genes from total DNA as well as DNA extracted from the heavy (containing ^13^C) CsCl fractions (buoyant density around 1.735 g mL^−1^) and *pmoA* gene were subjected to amplicon sequencing [59] using 515F/907R and A189f/mb661r primers, respectively. ^12^CH_4_-treated microcosms with similar buoyant density were also sequenced to compare contextual details.

Quantitative insights into microbial ecology (QIIME) pipeline key-quality control steps were applied to remove low quality sequences as previously described [59,60]. A total of 1,647,700 sequences from 16S rRNA genes of total DNA of both biofilms and 292,949 sequences of *pmoA* from the heavy fractions of both biofilms were obtained with a high-quality score of >20 without ambiguous base pairs and mismatched primers. The ribosomal data base (RDP) MultiClassifier was used to obtain taxonomic units at a similarity of 97% (or 3% cutoff) [61].

### 3.10. Statistical Analysis

Diversity indices such as operational taxonomic unit (OUT) richness, Chao1, Simpson index, Evenness index, and Shannon index were computed using the Vegan package in R 3.4.3 (https://www.reproject.org/) for both periphytic biofilms. PE data were subjected to statistical analysis of variance with the *p* < 0.05 indicating statistical significance (SPSS 20.0, Hong Kong, China).

## 4. Conclusions

Epiphyton and epixylon biofilms showed excellent methane oxidation, and Pb^2+^ and Cd^2+^ bioremediation along with PE biodegradation potential. After 20 days of incubation under high methane doses and chronic HMs and PE exposure, the individual and combined effects were measured based on HMs adsorption by epiphyton and epixylon. Treatments with 2 mg L^−1^ and 50 mg L^−1^ in both periphytic biofilms consumed all headspace methane along with HMs, but 100 mg L^−1^ only partially removed HMs. We found that the mechanism of HMs bioremediation, methane oxidation, and PE biodegradation is dependent on HM concentrations. The synergistic toxic effect of HMs and PE is more detrimental and enhanced than the discrete effect on methane oxidation and microbial communities. Methane oxidation enhanced PE biodegradation but the effect was reduced by the presence of HMs. In the presence of HMs and PE, the diversity and abundance of microbiota of specific phylotypes changed. *Methylobacter* sp. was dominant in all treatments, but the relative abundance in the presence of HMs and PE was lower. The relative abundance of *Methylococcus* increased. All the identified methanotrophic biota were enhanced in high-methane-dose treatments compared to NAM treatments. From the results of the current study, we conclude that epiphyton is more effective than epixylon, and both epiphyton and epixylon biofilms are ecofriendly and can be used for the remediation of multiple pollutants.

## Supplementary Materials

The following are available online at https://www.mdpi.com/1422-0067/21/15/5331/s1, Table S1. Structure and properties of polyethylene microplastic; Table S2. Experimental design for epiphyton containing metals, methane and polyethylene; Table S3. Experimental design for epixylon containing metals, methane and polyethylene; Table S4. Primers and PCR amplification conditions used in this study; Table S5. Molecular weight changes of polyethylene determined by GPC; Figure S1. Scanning Electron Microscopy at 10 μm of epiphyton and epixylon (a) SEM micrograph of epiphyton before treatment with 120000 ppm ^13^CH4 (b) SEM micrograph of epiphyton after treatment with 120000 ppm ^13^CH4 (c) AWCD and diversity indices of epiphyton (d) SEM micrograph of epixylon before treatment with 120000 ppm 13CH4 (e) SEM micrograph of epixylon after treatment with 120000 ppm ^13^CH4 (f) AWCD and diversity indices of epixylon. Thread like structures are algae and dead bacterial aggregates are clearly visible in after treatments; Figure S2. Percentage of ^13^C atoms abundance accumulated by methanotrophs under different methane, heavy metals doses along with polyethylene treatments (a) Epixylon ^13^C atom (%) assimilation (b) Epiphyton ^13^C atom (%) assimilation.
