# Supplementary material for: Multifunctional Periphytic Biofilms: Polyethylene Degradation and Cd2+ and Pb2+ Bioremediation under High Methane Scenario
Source: Int J Mol Sci. 2020 Jul 27;21(15):5331. doi: 10.3390/ijms21155331 (PMC7432609; doi:10.3390/ijms21155331)
Supplement: Supplementary file 1 [file ijms-21-05331-s001.pdf]

Multifunctional Periphytic Biofilms: Polyethylene Degradation and Cd^{2+} and Pb^{2+} Bioremediation under High Methane Scenario

Muhammad Faheem [1,2], Sadaf Shabbir [3], Jun Zhao [1], Philip G Kerr [4], Shafaqat Ali [5,7], Nasrin Sultana [1,2,6], and Zhongjun Jia [1,*]

[1] State Key Laboratory of Soil and Sustainable Agriculture, Institute of Soil Science, Chinese Academy of Sciences, Nanjing 210008, Jiangsu Province, China; faheem.shakoor@gmail.com (M.F.); zhaojun@issas.ac.cn (J.Z.); nasrinjc@issas.ac.cn (N.S.)

[2] University of Chinese Academy of Sciences, Beijing 100049, China

[3] College of Environment, Hohai University, 1 Xikang Road, Nanjing 210008, China; sadaf.dar83@gmail.com

[4] School of biomedical Science, Charles Sturt University, Wagga Wagga, NSW 2678, Australia; philip.kerr@gmail.com

[5] Department of Environmental Sciences and Engineering, Government College University, Faisalabad 38000, Pakistan; shafaqataligill@gcuf.edu.pk

[6] Department of Agroforestry and Environmental Science, Sher-e-Bangla Agricultural University (SAU), Sher-e-Bangla nagar, Dhaka 1207, Bangladesh

[7] Department of Biological Sciences and Technology, China Medical University, Taichung 40402, Taiwan

***** Correspondence: jia@issas.ac.cn

This SOM file contains:

Supplementary Table S1 to S5

Supplementary Figure S1 to S2

Supplementary References

Table S1. Molecular weight changes of polyethylene determined by GPC

**Treatments**	**M_w**	**M_n**	**M_w/M_n**
Control	182366	23321	7.81982
EPX + NAM + PE	173827	22322	7.78725
EPX + ^{13}C + PE	156834	19887	7.88626
EPX + ^{13}C + M1C2 + PE	162739	20823	7.81535
EPX + ^{13}C + M2C2 + PE	166723	21272	7.83767
EPP + NAM + PE	172345	21978	7.84171
EPP + ^{13}C + PE	152395	19626	7.76495
EPP + ^{13}C + M1C2 + PE	160345	20674	7.75588
EPP + ^{13}C + M2C2 + PE	163623	21078	7.76274

Table S2: Experimental design for epiphyton containing metals, methane and polyethylene

**Number of Treatments**	**Abbreviation**	**Treatment**
1	EPP + NAM	Epiphyton + Near Atmospheric Methane (6 ppm $^{13}CH_4$)
2	EPP + ^{12}C	Epiphyton + 120000 ppm $^{12}CH_4$
3	EPP + ^{13}C	Epiphyton + 120000 ppm $^{13}CH_4$
4	EPP + ^{13}C+ M1C1	Epiphyton + 120000 ppm $^{13}CH_4$ + Pb (2 mg/L)
5	EPP + ^{13}C + M1C2	Epiphyton + 120000 ppm$^{13}CH_4$ + Pb (50 mg/L)
6	EPP + ^{13}C + M1C3	Epiphyton + 120000 ppm $^{13}CH_4$ + Pb (100 mg/L)
7	EPP + ^{13}C + M2C1	Epiphyton + 120000 ppm $^{13}CH_4$ + Cd (2 mg/L)
8	EPP + ^{13}C + M2C2	Epiphyton + 120000 ppm $^{13}CH_4$ + Cd (50 mg/L)
9	EPP + ^{13}C + M2C3	Epiphyton + 120000 ppm $^{13}CH_4$ + Cd (100 mg/L)
10	EPP + NAM + PE	Epiphyton + Near Atmospheric Methane (6 ppm $^{13}CH_4$) + Polyethylene
11	EPP + ^{13}C + PE	Epiphyton + 120000 ppm $^{13}CH_4$ + Polyethylene
12	EPP + ^{13}C + M1C2 + PE	Epiphyton + 120000 ppm $^{13}CH_4$ + Pb (50 mg/L) + Polyethylene
13	EPP + ^{13}C + M2C2 + PE	Epiphyton + 120000 ppm $^{13}CH_4$ + Cd (50 mg/L) + Polyethylene

Table S3: Experimental design for epixylon containing metals, methane and polyethylene

Number of Treatments	Abbreviation	Treatment
1	EPX + NAM	Epixylon + Near Atmospheric Methane (6 ppm $^{13}CH_4$)
2	EPX + ^{12}C	Epixylon + 120000 ppm $^{12}CH_4$
3	EPX + ^{13}C	Epixylon + 120000 ppm $^{13}CH_4$
4	EPX + ^{13}C + M1C1	Epixylon + 120000 ppm $^{13}CH_4$ + Pb (2 mg/L)
5	EPX + ^{13}C + M1C2	Epixylon + 120000 ppm$^{13}CH_4$ + Pb (50 mg/L)
6	EPX + ^{13}C + M1C3	Epixylon + 120000 ppm $^{13}CH_4$ + Pb (100 mg/L)
7	EPX + ^{13}C + M2C1	Epixylon + 120000 ppm $^{13}CH_4$ + Cd (2 mg/L)
8	EPX + ^{13}C + M2C2	Epixylon + 120000 ppm $^{13}CH_4$ + Cd (50 mg/L)
9	EPX + ^{13}C + M2C3	Epixylon + 120000 ppm $^{13}CH_4$ + Cd (100 mg/L)
10	EPX + NAM + PE	Epixylon + Near Atmospheric Methane (6 ppm $^{13}CH_4$) + Polyethylene
11	EPX + ^{13}C + PE	Epixylon + 120000 ppm $^{13}CH_4$ + Polyethylene
12	EPX + ^{13}C + M1C2 + PE	Epixylon + 120000 ppm $^{13}CH_4$ + Pb (50 mg/L) + Polyethylene
13	EPX + ^{13}C + M2C2 + PE	Epixylon + 120000 ppm $^{13}CH_4$ + Cd (50 mg/L) + Polyethylene

Table S4. Primers and PCR amplification conditions used in this study

Name of primer	Sequence of primers (5'-3')	Target gene	Cycling conditions	Type of analysis	References
**515F**	GTGCCAGCMGCCGCGG	Universal bacterial 16S rRNA gene	95 °C, 5 min; 35× (95 °C, 30 s; 54 °C, 30 s; 72 °C, 30 s); 72 °C, 8 min	Illumina MiSeq sequencing	(Stubner, 2002)
**907R**	CCGTCAATTCMTTTRAGTTT				
**A189F**	GGNGACTGGGACTTCTGG	Methanotrophic *pmoA* gene	95 °C, 3 min; 33× (95 °C,10 s; 54 °C, 30 s; 72 °C, 30 s; 80 °C, 5 s; plate read); melt curve 65 °C to 95 °C, incremental 0.5 °C, 0:05+plate read	Real-time qPCR	(Costello and Lidstrom, 1999; Holmes et al., 1995)
**mb661r**	CCGGMGCAACGTCYTTACC		95 °C,5 min; 33× (95 °C,30s; 54 °C, 30 s; 72 °C, 45 s); 72 °C, 10 min	High throughput MiSeq sequencing	

Table S5: Structure and properties of polyethylene microplastic

Type	Specific gravity	Structure	Production 2020	Use/Application	Reference
**Polyethylene**	0.91–0.96	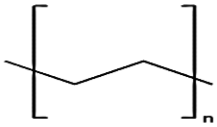	3.3 million tones	Extensively used in huge industrial production of plastic bags and plastic bottles	(Shabbir et al., 2020)

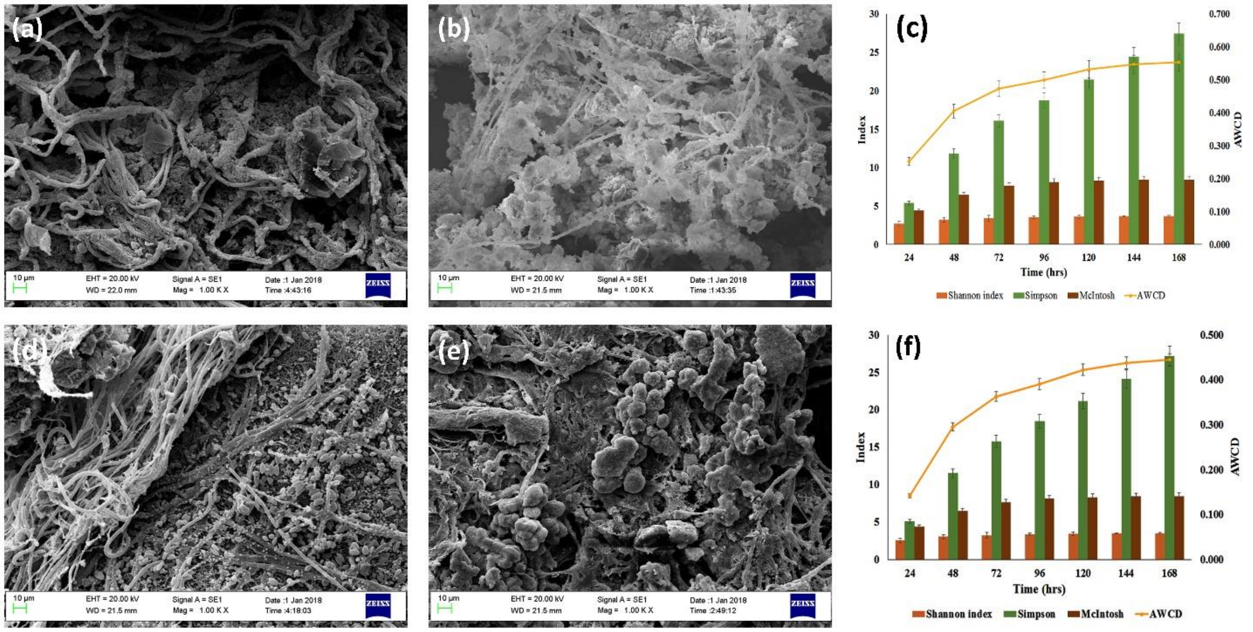

Figure S1: Scanning Electron Microscopy at 10 μm of epiphyton and epixylon (a) SEM micrograph of epiphyton before treatment with 120000 ppm $^{13}CH_4$ (b) SEM micrograph of epiphyton after treatment with 120000 ppm $^{13}CH_4$ (c) AWCD and diversity indices of epiphyton (d) SEM micrograph of epixylon before treatment with 120000 ppm $^{13}CH_4$ (e) SEM micrograph of epixylon after treatment with 120000 ppm $^{13}CH_4$ (f) AWCD and diversity indices of epixylon. Thread like structures are algae and dead bacterial aggregates are clearly visible in after treatments.

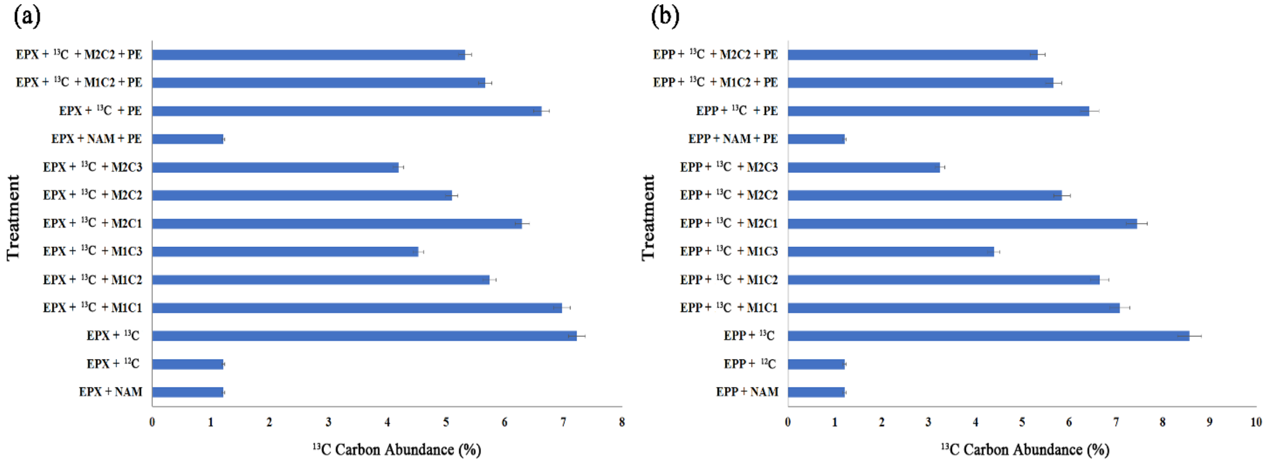

Figure S2: Percentage of ^{13}C atoms abundance accumulated by methanotrophs under different methane, heavy metals doses along with polyethylene treatments (a) Epixylon ^{13}C atom (%) assimilation (b) Epiphyton ^{13}C atom (%) assimilation.

References

Costello AM, Lidstrom ME. Molecular characterization of functional and phylogenetic genes from natural populations of methanotrophs in lake sediments. Applied and environmental microbiology 1999; 65: 5066-5074.

Holmes AJ, Costello A, Lidstrom ME, Murrell JC. Evidence that particulate methane monooxygenase and ammonia monooxygenase may be evolutionarily related. FEMS Microbiol Lett 1995; 132: 203-8.

Shabbir S, Faheem M, Ali N, Kerr PG, Wang L-F, Kuppusamy S, et al. Periphytic biofilm: An innovative approach for biodegradation of microplastics. Science of The Total Environment 2020; 717: 137064.

Stubner S. Enumeration of 16S rDNA of Desulfotomaculum lineage 1 in rice field soil by real-time PCR with SybrGreen™ detection. Journal of Microbiological Methods 2002; 50: 155-164.
